# Public Awareness of Dupuytren’s Contracture in Saudi Arabia

**DOI:** 10.7759/cureus.78695

**Published:** 2025-02-07

**Authors:** Yosra F Buhiliga, Hussam F Alkhars, Abdullah Alkhars, Abdullah AlAlwan, Ali F Alkhars

**Affiliations:** 1 Plastic and Reconstructive Surgery, Dammam Medical Complex, Dammam, SAU; 2 Medicine and Surgery, King Faisal University, Al Hofuf, SAU; 3 Orthopedics, King Fahad Hospital, Al Hofuf, SAU; 4 Plastic and Reconstructive Surgery, King Faisal University, Al Hofuf, SAU; 5 Medicine, King Faisal University, Al Hofuf, SAU

**Keywords:** awareness, dupuytren’s contracture, knowledge, public awareness, saudi arabia

## Abstract

Introduction

Dupuytren’s contracture (DC) is a progressive and irreversible fibroproliferative disorder affecting the palmar fascia. Restricting hand mobility can significantly impact an individual’s quality of life. This study aims to assess public awareness of DC in Saudi Arabia and identify factors associated with awareness levels to inform prevention and management strategies.

Materials and methods

A cross-sectional study was conducted from January to October 2024 among Saudi nationals aged 18 years and older. Data were collected via a Google Forms (Google LLC, Mountain View, CA, USA) survey distributed on social media platforms. The questionnaire was validated using the Lawshe method, and knowledge of DC symptoms, risk factors, and treatment options was assessed. Statistical analyses were performed using SPSS Statistics version 26.0 (IBM Corp. Released 2019. IBM SPSS Statistics for Windows, Version 26.0. Armonk, NY: IBM Corp.). Participants were categorized into “poor” and “good” knowledge groups based on a knowledge score. Factors associated with knowledge and awareness were analyzed, with p-values <0.05 considered statistically significant.

Results

A total of 713 participants were included in the study. Awareness of DC was generally low, with 231 participants (32.4%) having heard of the condition. Only 28.3% identified diabetes as a risk factor, and 28.5% recognized finger immobility as a symptom. Treatment awareness was limited; while 28.2% knew about surgical options, fewer participants knew of non-surgical treatments such as collagenase injections (24.5%) and physical therapy (29.4%). Factors significantly associated with better awareness included residing in the Central Region (p<0.001), being aged 36-45 years (p<0.001), holding postgraduate degrees (p<0.001), and having previous knowledge of DC (p=0.00001).

Conclusions

Public knowledge and awareness of DC in Saudi Arabia were limited, with significant gaps in understanding its risk factors, symptoms, and treatment options. Educational campaigns are needed to improve awareness, particularly regarding the associations with diabetes, family history, and lifestyle factors such as tobacco and alcohol use. Increasing public knowledge of clinical symptoms and treatment options could lead to earlier diagnosis and better management. Further research is recommended to explore regional and population-level differences in awareness and to address limitations in understanding DC.

## Introduction

Dupuytren’s contracture (DC), also known as Dupuytren's disease, was first described in 1614 by Felix Platter, according to Peimer et al. [1]. It is classified as a fibroproliferative disorder characterized by developing collagen fibers in the palmar fascia [1-3]. DC is a slowly progressive and irreversible condition. Fibrotic changes typically begin in the palm and extend to the fingers, most commonly affecting the ring finger, followed by the little finger [4]. Although not painful, the disease causes progressive restriction of movement in the affected digits as it advances [4,5].

The exact cause of DC remains unclear. However, immune cells in affected areas suggest a possible link to the immune system [5]. Numerous studies have also identified significant associations with environmental and genetic factors [4,6]. Environmental risk factors include smoking, alcohol consumption, hand and finger injuries, and vigorous physical activities involving the hands [7,8]. Although not considered life-threatening, DC can significantly impact an individual’s quality of life, affecting daily activities such as washing, picking up, and holding objects [9,10].

Research exploring public awareness and understanding of this condition is limited. Gaining insight into community perceptions and knowledge of DC is essential for improving patient outcomes and public awareness. This study aims to address this gap by investigating the knowledge and understanding of DC among the Saudi Arabian population.

## Materials and methods

Study design

We conducted a community-based cross-sectional study from January to October 2024 in all regions of Saudi Arabia.

Population and sample size

The study targeted the entire population of Saudi Arabia. Using an online Raosoft sample size calculator (http://www.raosoft.com/samplesize.html), the required sample size was calculated to be 385 with an interval of confidence at 95%, a rate of response at 50%, and a 5% margin of error.

Inclusion and exclusion criteria

The inclusion criteria encompassed Saudi Arabian inhabitants aged 18 years and older. The exclusion criteria included those not residing in Saudi Arabia.

Data collection

The study’s questionnaire was initially designed in English and subsequently translated into Arabic to facilitate participation and comprehension. The questionnaire’s validity was assessed using the Lawshe method. A panel of 12 experts in the field reviewed the questionnaire, and the content validity ratio (CVR) was calculated. Items with a CVR below 0.99 were removed. Reliability was evaluated through a pilot study involving 211 participants; data from the pilot study were excluded from the final analysis. Participant privacy was safeguarded throughout the study. Ethical approval was obtained from the Research Ethics Committee of King Faisal University (reference code: KFU-REC-2024-NOV-ETHICS2921).

We used a non-probability convenience sampling to invite participants who met the inclusion and exclusion criteria. The online questionnaire was distributed via all social media platforms as a Google Form (Google LLC, Mountain View, CA, USA). The distributed questionnaire was written in Arabic for better understanding and clarity for the Saudi participants and was divided into two subscales. The first subscale focused on the demographic data of the participants. The second subscale focused on the participant's general awareness and knowledge of DC, including previous knowledge, risk factors, symptoms, and management options.

Data was collected using a Google Forms survey distributed via social media platforms. For initial organization, responses were compiled in Microsoft Excel (Microsoft Corporation, Redmond, WA, USA).

Statistical analysis

Statistical analyses were performed using SPSS Statistics version 26.0. (IBM Corp. Released 2019. IBM SPSS Statistics for Windows, Version 26.0. Armonk, NY: IBM Corp.). Categorical variables were summarized as frequencies and percentages to describe sample characteristics. A knowledge score was created by awarding one point for each correct answer to knowledge-related questions. Participants were categorized into two groups: those with “poor” knowledge (scoring less than 60%) and those with “good” knowledge (scoring 60% or higher). The Chi-square test was used to test the associations between these categorical variables and the knowledge level (poor vs. good). A p-value of less than 0.05 was considered statistically significant.

## Results

Sociodemographic characteristics

Table 1 presents the sociodemographic characteristics of the study participants (n=713). Participants were fairly distributed across regions, with the Central Region (184 participants, 25.8%) and the Western Region (172 participants, 24.1%) having the highest representation. The Southern Region had the smallest proportion (78 participants, 10.9%). Regarding age distribution, most participants were in the 26-35 years (178 participants, 25.0%) and 46-55 years (175 participants, 24.5%) age groups. More than half of the participants were men (378 participants, 53.0%), while 335 (47.0%) were women.

**Table 1:** Sociodemographic characteristics of study participants in Saudi Arabia (N=713) The data are represented as (N) and (%) for participants in each question. SR: Saudi Riyals

Sociodemographic data		N	Percentage
Residence region	Central region	184	25.8%
	Northern region	113	15.8%
	Eastern region	166	23.3%
	Western region	172	24.1%
	Southern region	78	10.9%
Age in years	18-25	97	13.6%
	26-35	178	25.0%
	36-45	169	23.7%
	46-55	175	24.5%
	>55	94	13.2%
Gender	Male	378	53.0%
	Female	335	47.0%
Educational level	Not educated	68	9.5%
	General education	68	9.5%
	Diploma	126	17.7%
	University graduate	154	21.6%
	Postgraduate degree	297	41.7%
Work status	Not working	171	24.0%
	Student	164	23.0%
	Employee	378	53.0%
Monthly income	<5000 SR	119	16.7%
	5000-10000 SR	215	30.2%
	10000-20000 SR	246	34.5%
	>20000 SR	133	18.7%
Marital status	Single	242	33.9%
	Married	352	49.4%
	Divorced/widowed	119	16.7%

In terms of educational attainment, 297 participants (41.7%) held postgraduate degrees and 154 (21.6%) were university graduates. Employment status data revealed that 378 participants (53.0%) were employed, 171 (24.0%) were not working, and 164 (23.0%) were students. Monthly income distribution indicated that most participants fell within the middle-income category, with 246 participants (34.5%) earning 10,000-20,000 Saudi Riyals (SR) and 215 (30.2%) earning 5,000-10,000 SR. Additionally, 119 participants (16.7%) reported incomes of less than 5,000 SR, while 133 (18.7%) earned more than 20,000 SR. Regarding marital status, nearly half of the participants (352 participants, 49.4%) were married, while 242 (33.9%) were single, and 119 (16.7%) were divorced or widowed.

Public knowledge and awareness

Table 2 summarizes the public knowledge and awareness of DC among participants in Saudi Arabia (n=713). Overall, 231 participants (32.4%) reported prior awareness of DC. However, knowledge of specific risk factors was limited, with 202 participants (28.3%) recognizing diabetes as a risk factor and 219 (30.7%) linking tobacco and alcohol use to the condition. Additionally, 192 participants (26.9%) knew that a medical history could increase the risk of DC.

**Table 2:** Public knowledge and awareness of DC in Saudi Arabia (N=713) The data are represented as (N) and (%) for participants in each question. DC: Dupuytren’s contracture

Domain	Knowledge items	Answer	N	Percentage
Previous knowledge	Have you ever heard of DC?	Yes	231	32.4%
		No	482	67.6%
Risk factors	Which of the following age groups increases the risk of developing DC?	1-20 years	112	15.7%
		21-30 years	206	28.9%
		31-50 years	233	32.7%
		>50 years	162	22.7%
	Which of the following is the risk of developing DC increased?	Male	360	50.5%
		Female	353	49.5%
	People with diabetes are more likely to develop DC.	Yes	202	28.3%
		No	218	30.6%
		I don't know	293	41.1%
	The risk of developing DC increases if there is a family medical history.	Yes	192	26.9%
		No	243	34.1%
		I don't know	278	39.0%
	People who use tobacco and alcohol are more likely to develop DC.	Yes	219	30.7%
		No	215	30.2%
		I don't know	279	39.1%
Symptoms	One of the symptoms of DC is a hard lump in the palm that strongly pulls the fingers toward it.	Yes	196	27.5%
		No	191	26.8%
		I don't know	326	45.7%
	One of the symptoms of DC is the inability to move the fingers and their sticking toward the palm.	Yes	203	28.5%
		No	192	26.9%
		I don't know	318	44.6%
	DC usually affects the two fingers furthest from the thumb.	Yes	164	23.0%
		No	167	23.4%
		I don't know	382	53.6%
Management	DC can be treated with physical therapy and acupuncture.	Yes	210	29.4%
		No	208	29.2%
		I don't know	295	41.4%
	DC can be treated with cortisone and collagenase injections.	Yes	175	24.5%
		No	158	22.2%
		I don't know	380	53.3%
	DC can be treated surgically.	Yes	201	28.2%
		No	162	22.7%
		I don't know	350	49.1%

Awareness of symptoms was also low; only 196 participants (27.5%) correctly identified a hard lump in the palm pulling the fingers, and 203 participants (28.5%) recognized finger immobility as a characteristic feature. Furthermore, only 164 participants (23.0%) knew that DC most commonly affects the ring finger, followed by the little finger. Regarding treatment options, 201 participants (28.2%) identified surgery as a treatment, while 210 (29.4%) and 175 (24.5%) were aware of physical therapy and collagenase injections, respectively. Most participants (509 participants, 71.4%) demonstrated poor knowledge and awareness of DC, while only 204 participants (28.6%) exhibited good knowledge.

Overall public knowledge and awareness

**Figure 1:**
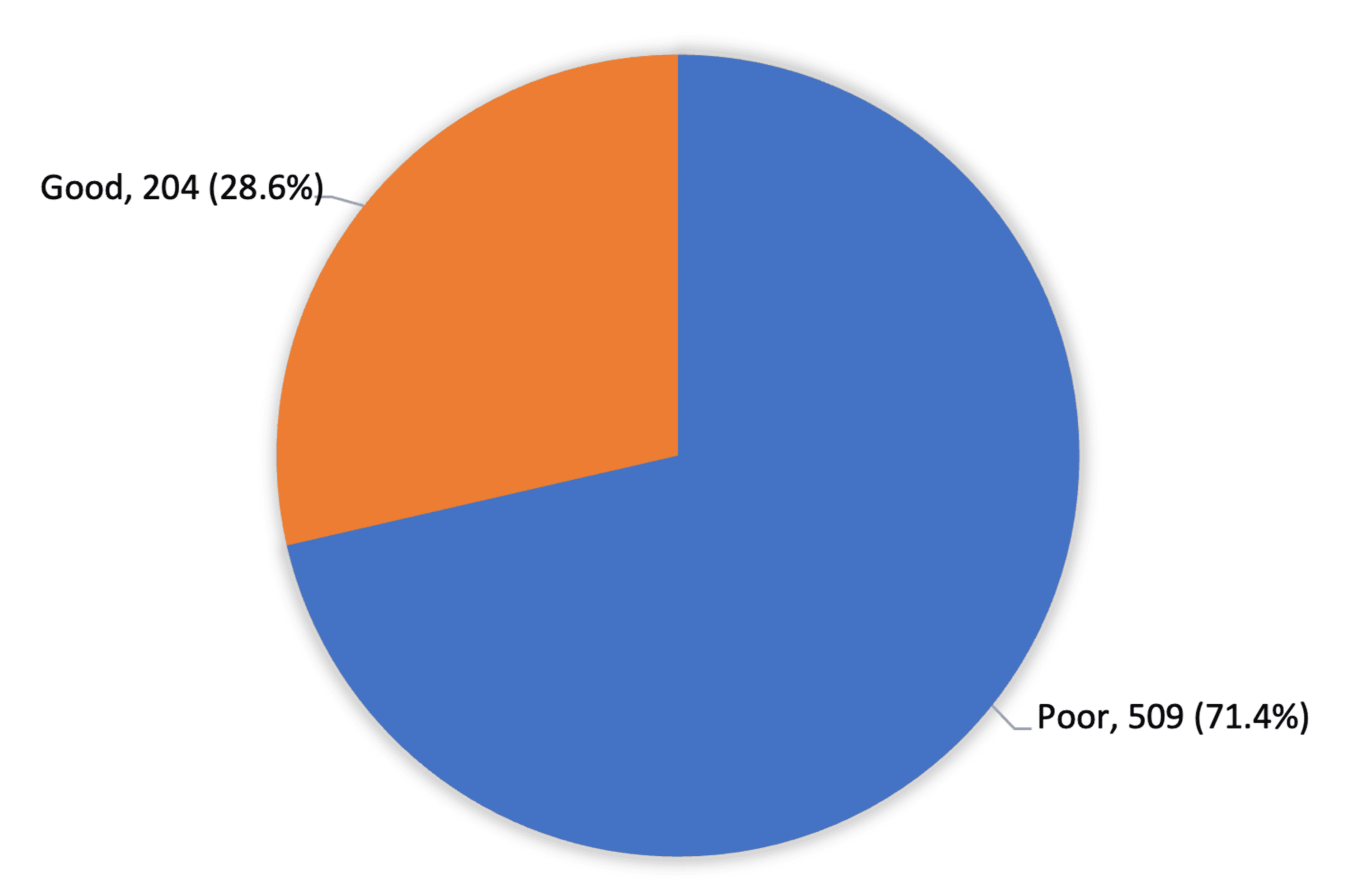
Overall public knowledge and awareness of DC in Saudi Arabia (n=713) The data are represented as (N) and (%) for the overall score of both categories. DC: Dupuytren’s contracture

Regarding the overall knowledge and awareness level (Figure 1), the vast majority of the participants (509, 71.4%) had an overall poor knowledge and awareness of DC, and only 204 (28.6%) had a good knowledge level.

Factors associated with knowledge and awareness

Table 3 outlines factors associated with knowledge and awareness of DC. Participants from the Central Region demonstrated the highest proportion of good knowledge and awareness (77 participants, 41.8%). In comparison, those from the Eastern Region had the lowest (37 participants, 22.3%), with a statistically significant difference (p<0.001).

**Table 3:** Factors associated with participants' knowledge and awareness of DC in Saudi Arabia (N=713) ^a ^Pearson X^2^ test, * statistically significant (p<0.05) SR: Saudi Riyals, DC: Dupuytren’s contracture

Factors		Overall knowledge and awareness level				P-value^a^
		Poor		Good		
		N	%	N	%	
Residence region	Central region	107	58.2%	77	41.8%	0.00001*
	Northern region	97	85.8%	16	14.2%	
	Eastern region	129	77.7%	37	22.3%	
	Western region	123	71.5%	49	28.5%	
	Southern region	53	67.9%	25	32.1%	
Age in years	18-25	75	77.3%	22	22.7%	0.000343*
	26-35	136	76.4%	42	23.6%	
	36-45	101	59.8%	68	40.2%	
	46-55	120	68.6%	55	31.4%	
	>55	77	81.9%	17	18.1%	
Gender	Male	259	68.5%	119	31.5%	0.072
	Female	250	74.6%	85	25.4%	
Educational level	Not educated	56	82.4%	12	17.6%	0.000646*
	General education	53	77.9%	15	22.1%	
	Diploma	103	81.7%	23	18.3%	
	University graduate	105	68.2%	49	31.8%	
	Postgraduate degree	192	64.6%	105	35.4%	
Work status	Not working	134	78.4%	37	21.6%	0.00398*
	Student	125	76.2%	39	23.8%	
	Employee	250	66.1%	128	33.9%	
Monthly income	<5000 SR	92	77.3%	27	22.7%	0.002741*
	5000-10000 SR	151	70.2%	64	29.8%	
	10000-20000 SR	187	76.0%	59	24.0%	
	>20000 SR	79	59.4%	54	40.6%	
Marital status	Single	202	83.5%	40	16.5%	0.00001*
	Married	243	69.0%	109	31.0%	
	Divorced/widowed	64	53.8%	55	46.2%	
Have you ever heard of DC?	Yes	48	20.8%	183	79.2%	0.00001*
	No	461	95.6%	21	4.4%	

Age was also significantly associated with knowledge levels (p<0.001), with participants aged 36-45 years showing the highest percentage of good knowledge (68 participants, 40.2%) and those aged above 55 years showing the lowest (17 participants, 18.1%). Men exhibited slightly higher awareness (119 participants, 31.5%) compared to women (85 participants, 25.4%), though this difference was not statistically significant (p=0.072).

Participants with postgraduate degrees demonstrated the highest knowledge levels (105 participants, 35.4%), with significant differences observed across educational levels (p<0.001). Employment status and monthly income significantly influenced knowledge levels (p<0.05 for each). Marital status was also significantly associated with knowledge and awareness (p<0.001). Notably, participants with previous knowledge of DC were more likely to exhibit good knowledge and awareness (183 participants, 79.2%) compared to those without previous knowledge (21 participants, 4.4%; p<0.05).

## Discussion

This study assessed public knowledge and awareness of DC in Saudi Arabia and identified factors associated with awareness levels. The findings highlight a lack of public awareness and knowledge regarding DC among the Saudi Arabian population, with only 204 participants (28.6%) displaying good knowledge compared to 509 participants (71.4%) who had poor knowledge. According to a previous study by Benson et al. that explored DC in depth regarding risk factors [11], public knowledge of these risk factors was generally poor. Only 162 participants (22.7%) out of 713 recognized that individuals above 50 years of age are at higher risk of developing DC than younger individuals. On the other hand, a slightly higher percentage correctly identified that males (360 participants, 50.5%) were at higher risk than females. Additionally, significant risk factors such as previous family medical history, diabetes mellitus, tobacco use, and alcohol abuse were poorly recognized. Awareness regarding treatment options was also low. Only 210 participants (29.4%) identified physical therapy and acupuncture as treatment methods, while cortisone and collagenase injections were recognized by 175 participants (24.5%). Surgical intervention was acknowledged by 201 participants (28.2%). These findings suggest a poor awareness level, aligning with a previous study highlighting surgical and non-surgical treatments' effectiveness in managing DC [12].

Several factors in this study contributed to good awareness and knowledge of DC, including residency in the Central region, middle age, postgraduate education, employment, high monthly income, marital status, and prior knowledge of the disease. However, overall public awareness of DC remained poor, highlighting significant gaps in understanding. Although 32.4% of participants had heard of the condition, detailed knowledge was lacking. Awareness of risk factors, such as diabetes, family history, and tobacco or alcohol use, was particularly low. This lack of understanding may delay diagnosis and management, emphasizing the need for targeted educational campaigns to address these gaps. Awareness of DC symptoms was similarly limited, with fewer participants recognizing the characteristic hard lump in the palm that pulls the fingers inward. Knowledge of treatment options was also inadequate. At the same time, some participants correctly identified surgical management, but fewer were aware of less invasive treatments such as cortisone injections, collagenase injections, physical therapy, and acupuncture. These findings align with studies by Lanting et al. [13] and the Dupuytren Research Group [14], which reported lower awareness of DC in regions outside Europe and North America. Despite ongoing public health campaigns, gaps in public knowledge about DC persist, particularly in areas where the condition is less common [15].

This study provides valuable insights into public knowledge and awareness of DC in Saudi Arabia; however, several limitations should be considered. Using social media to distribute the questionnaire may have introduced selection bias, potentially excluding individuals without internet access and skewing the sample toward younger, tech-savvy participants. Additionally, the uneven regional representation limits the generalizability of the findings, as some areas were underrepresented. The reliance on self-reported data raises the possibility of recall and response bias, which may affect the accuracy of reported knowledge and awareness. Furthermore, the cross-sectional design restricts causal interpretations, meaning that associations identified between demographic factors and DC cannot confirm causation. Lastly, the study did not examine cultural or healthcare access factors that could influence awareness, nor did it assess the quality or sources of participants’ information.

## Conclusions

This study assessed public knowledge and awareness of DC in Saudi Arabia and identified factors influencing awareness levels. The results revealed a significant gap in the population’s understanding of DC, with many individuals unaware of its risk factors, symptoms, and treatment options. Public education campaigns are urgently needed to raise awareness, particularly regarding the associations between DC and diabetes, family history, and lifestyle factors such as tobacco and alcohol use. Increased awareness of clinical symptoms and available surgical and non-surgical treatments could lead to earlier diagnosis and improved management of the condition. Further research on public awareness and knowledge of DC is recommended to explore the factors contributing to regional and population-level differences in awareness. Enhancing public education and awareness about DC is crucial for effective prevention, early detection, and optimal management of the condition.
